# Postoperative radiotherapy-induced pulmonary leiomyosarcoma in breast cancer: a case report and literature review

**DOI:** 10.3389/fonc.2026.1831335

**Published:** 2026-06-12

**Authors:** Dewei Zhang, Shihan Shi, Yang Wei, Jin Wei, Linwei Yang, Si Gui, Xuejian Wang, Liming Zhou, Yan Zhang

**Affiliations:** 1 Department of Medical Imaging, Guihang 300 Hospital Affiliated to Zunyi Medical University, Guiyang, China; 2 Guizhou Medical University, Guiyang, China

**Keywords:** breast cancer, imaging findings, leiomyosarcoma, radiation-associated sarcomas, radiotherapy

## Abstract

Radiation-induced sarcoma (RIS) is a rare and severe complication of radiotherapy following breast cancer surgery. It is characterized by a long latency period, high malignancy, limited clinical experience in diagnosis and treatment, and substantial individual variation in prognosis. This study reports a case of radiation-related pulmonary leiomyosarcoma that developed more than 20 years after radiotherapy for breast cancer. The tumor harbored a TP53 mutation and showed low PD-L1 expression. Imaging documented rapid tumor progression over a short interval, which rendered radical resection impossible and complicated efforts to improve quality of life. However, the patient attempted to receive multiple treatments—interventional therapy, chemotherapy, targeted therapy, and immunotherapy—but the response was poor, survival was under 10 months, and the patient ultimately died.

## 1 Introduction

Radiation therapy (RT) is a key modality in cancer treatment, with more than 50% of cancer patients requiring RT (1). Although radiotherapy can improve long-term survival, it may also induce secondary tumors due to the carcinogenic effects of ionizing radiation (2). Studies have shown that postoperative radiotherapy after radical mastectomy for breast cancer significantly reduces the risk of local recurrence but also increases the incidence of late complications of breast cancer radiotherapy, such as RIS (3). Although postoperative RIS in breast cancer, which is an uncommon and serious complication, has been reported, its high malignancy and limited clinical diagnosis and treatment experience lead to large individual differences in prognosis (4). We herein present a rare case of pulmonary leiomyosarcoma occurring more than 20 years after adjuvant radiotherapy for breast cancer, with molecular characterization revealing a TP53 mutation and low Programmed deathligand 1 (PD-L1) expression. The disease’s refractory course and minimal response to multimodal therapy further emphasize the substantial therapeutic challenges associated with this condition. Although the patient ultimately died, this case offers important clinical insights and valuable references for improving the quality of life for patients with similar circumstances.

## 2 Case presentation

An 83-year-old woman was diagnosed with breast cancer at the Affiliated Hospital of Guizhou Medical University more than two decades ago. She underwent a radical mastectomy followed by radiotherapy and chemotherapy. Regular follow-up exams showed no evidence of cancer recurrence or spread. Recently, she developed respiratory symptoms after a cold, including cough, white phlegm, fever and gradually experienced poor appetite and reduced activity that affected her daily life. Initially, her condition improved with oral medications, but it worsened five days later. A non-contrast chest computed tomography (CT) on December 30, 2022, revealed infectious lung lesions consistent with a novel coronavirus-related pulmonary infection. Additionally, a 38 mm × 48 mm mass was found in the upper right lung lobe near the mediastinum, with bone destruction of adjacent ribs and mildly enlarged mediastinal and left axillary lymph nodes (**Figures 1A–C**). A follow-up contrast-enhanced chest CT performed 29 days after the initial study showed the mass had grown to 52 mm × 58 mm (**Figure 1D**). Laboratory tests indicated carbohydrate antigen 125 was 243 U/mL (normal range: 0–25 U/mL) and carbohydrate antigen 72–4 was 9.75 U/mL (normal range: 0-6.9 U/mL). Therefore, a biopsy was recommended for a definitive diagnosis.

**Figure 1:**
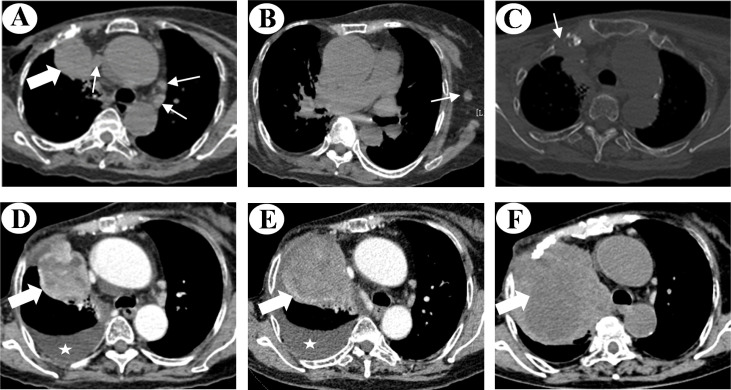
**(A–C)** Axial non-contrast chest CT shows a right upper-lobe mass (thick arrow), bone destruction of adjacent ribs (thin arrow), and enlargement of mediastinal and left axillary lymph nodes (thin arrow). The right breast is absent. **(D–F)** Follow-up axial contrast-enhanced chest CT shows progressive enlargement of the right upper-lobe mass with pronounced heterogeneous enhancement after contrast administration (thick arrow). A small right pleural effusion is also present (star).

Seventeen days later, a contrast-enhanced chest CT showed further enlargement of the lesion (**Figure 1E**). After contraindications to percutaneous aspiration biopsy were excluded, the patient underwent CT-guided percutaneous aspiration biopsy of the mass. Microscopically, the lesion showed a malignant mesenchymal tumor mainly composed of spindle-shaped cells, arranged in intersecting fascicles, with marked nuclear atypia, elongated hyperchromatic nuclei, eosinophilic cytoplasm, visible atypical mitoses and focal tumor necrosis (**Figure 2A**). Immunohistochemical (IHC) staining was positive for EGFR, Vimentin, EMA, P120, SMA and Desmin. SMA was positive in 70–80% of neoplastic cells with moderate-to-strong cytoplasmic staining intensity, and Desmin was positive in 80% of neoplastic cells with moderate-to-strong cytoplasmic staining intensity (**Figure 2B**). The Ki-67 proliferation index was approximately 20%. The tumor cells were negative for ER, PR, CerbB-2, CK, CK20, Villin, CK5/6, CgA, CK7, NapsinA, TTF-1, HMB45, CD34, MelanA, Syn, S-100, CD56, SOX-10, MyoD1, Myogenin and E-cadherin. Genetic testing showed the TP53 mutation with 68% variant allele frequency, PD-L1 Tumor Proportion Score (TPS) of 1%–2%, and a Combined Positive Score (CPS) of 3. In summary, the case was diagnosed as postoperative radiotherapy–associated pulmonary leiomyosarcoma of the breast cancer.

**Figure 2:**
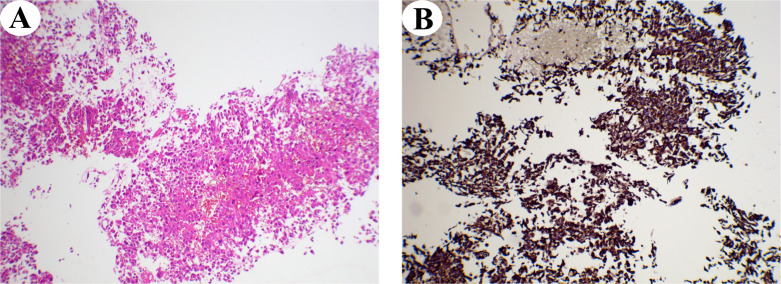
Representative histologic images of radiation-induced pulmonary leiomyosarcoma. **(A)** Malignant mesenchymal tumor composed mainly of spindle-shaped cells arranged in intersecting fascicles, with marked nuclear pleomorphism, hyperchromatic nuclei, eosinophilic cytoplasm, atypical mitoses and focal tumor necrosis (H&E, ×200). **(B)** Desmin (80% +) by immunohistochemistry (H&E, ×200).

After a multidisciplinary discussion, surgical resection was considered not feasible because the mass was large, the patient was elderly with poor physical tolerance, and she declined invasive surgery due to her advanced age and physical frailty. 10 days later, the patient underwent transcatheter arterial chemoembolization (TACE). 11 days after that, the patient received systemic intravenous chemotherapy with doxorubicin and ifosfamide, but experienced severe nausea and vomiting, significantly impairing her quality of life and preventing her from tolerating further aggressive anti-tumor treatment. 3 days later, oral anlotinib was administered for antitumor treatment. 20 days later, the patient received camrelizumab immunotherapy and, 4 days later, underwent TACE again. Five months after the initial examination, contrast-enhanced chest CT re-evaluation showed no remission (**Figure 1F**). Instead, the tumor had increased by more than twofold. This result was clearly discouraging. Given the poor tumor response and the patient’s declining physical status, both the patient and her family agreed to receive palliative symptomatic care to relieve symptoms and maintain comfort, rather than continuing empirical multi-line treatment. Unfortunately, the patient survived less than 10 months before dying. **Table 1** clearly illustrates the clinical timeline of the patient’s disease course.

**Table 1:** Clinical timeline of the patient’s disease course.

Time node	Key clinical events
>20 years prior	Diagnosed with breast cancer; received radical mastectomy plus postoperative radiotherapy and chemotherapy for breast cancer
Dec 2022	Presented with cough, sputum and fever; chest CT revealed right upper pulmonary mass complicated with pulmonary infection
Jan 2023	Chest−enhanced CT showed progressive enlargement of the pulmonary mass; CT−guided percutaneous lung biopsy confirmed radiation−induced pulmonary leiomyosarcoma
Late Jan 2023	MDT discussion was performed; surgical resection was abandoned, and the patient received the first TACE
11 days after first TACE	Received systemic chemotherapy with doxorubicin combined with ifosfamide
3 days after chemotherapy	Severe gastrointestinal adverse reactions occurred; switched to oral anlotinib targeted therapy
20 days after targeted therapy	Received sequential camrelizumab immunotherapy
4 days after immunotherapy	Underwent the second TACE
5 months after initial diagnosis	Re−examined chest CT showed tumor progression
Within 10 months after initial diagnosis	The patient suffered continuous disease deterioration and died

## 3 Discussion

Radiation-induced sarcoma (RIS) has a low incidence, and less than 1% of patients receiving radiotherapy develop radiation-associated sarcoma (5). However, it is a non-negligible complication among long-term surviving breast cancer patients. Literature indicates that breast cancer is the most common primary malignant tumor associated with RIS, with lymphoma ranking second (2, 6, 7). The most common histological types of RIS are angiosarcoma and undifferentiated pleomorphic sarcoma, followed by malignant peripheral nerve sheath tumor, leiomyosarcoma, and osteosarcoma (2, 6, 8). The diagnostic criteria for RIS, as revised by Cahan (9), include: (1) a documented history of radiotherapy; (2) sarcoma developing within the irradiated field; (3) no tumor was present before radiotherapy; (4) a latency period of at least 6 months after radiotherapy before the sarcoma appears; and (5) a new tumor confirmed by pathological diagnosis, histologically distinct from the primary tumor. Diagnosing leiomyosarcoma also involves pathology and immunohistochemistry, demonstrating smooth muscle differentiation, immunoreactivity for SMA, Desmin, and Vimentin, and negativity for CK. Some cases may also express EMA and S-100 (4, 10). The present case meets these diagnostic criteria.

To date, no studies have compared and analyzed cases of radiotherapy-induced leiomyosarcoma in breast cancer patients. This study presents a comprehensive review of such cases from recent English-language literature, and the results are summarized in **Table 2**. The analysis included key factors such as age at onset, latency period, site of occurrence, TP53 and PD-L1 status, treatment regimens, and prognosis. Among the 7 cases with follow-up information, 5 cases achieved long-term survival without tumor recurrence after radical resection, while 1 case still experienced recurrence, and another case died during the second chemotherapy cycle after chemotherapy. Additionally, among the 2 cases with TP53 gene mutations, 1 case achieved survival without tumor recurrence after radical resection, but unfortunately, the follow-up time data was missing. The other case was a patient diagnosed with Li-Fraumeni syndrome, and its follow-up records were also unavailable. The latent periods of the 9 cases ranged from 2 to 20 years, and most tumors arose in the breast and chest wall within the original radiotherapy field. It is rarely reported that our case occurred in the lung within the radiotherapy field, and the latent period exceeded 20 years, which further supplemented the clinical data of this case. Moreover, there is currently no reported case of radiotherapy-related leiomyosarcoma after breast cancer surgery with both TP53 mutation and low PD-L1 expression. The optimal therapeutic strategy for such molecular phenotype remains undefined. Although the present case ended in failure, it provides important clinical warnings and practical references for clinicians. For patients identified early with unique therapy-resistant molecular phenotypes, withholding empirical multi-line treatment may help better preserve long-term quality of life.

**Table 2:** The clinicopathological characteristics of radiation-induced leiomyosarcoma after breast cancer radiotherapy.

No.	Cases	Patient profile	Latent period	Tumor location	TP53 mutation	PD-L1 status	Treatment	Follow-up
1	Sakurai et al. (11)	66 years, F	18 years	Breast skin	NA	NA	Resection	5 years, ANED
2	Olcina et al. (12)	57 years, F	20 years	Breast area	NA	NA	Chemo	Died after second course
3	Henry et al.* (13)	24 years, F	2.2 years	Chest wall	Yes	NA	Chemo+RT	NA
4	Hayashi et al. (14)	59 years, F	6 years	Breast	NA	NA	Resection	20 months, ANED
5	Petry et al. (15)	52 years, F	15.7 years	Chest wall	Yes	NA	Resection	ANED
6	Liu et al. (4)	28 years, F	2 years	Chest wall	No	NA	Resection	1 year, ANED
7	Badagionis et al. (16)	83 years, F	5 years	Chest wall	NA	NA	Resection	NA
8	Zhang et al. (10)	45 years, F	5 years	Breast+Chest wall	NA	NA	Resection	13 months, Recurrence
9	Negishi et al. (17)	60 years, F	11.7 years	Breast	NA	NA	Resection	89 months, ANED

F, female; ANED, alive with no evidence of disease; NA, not available; RT, radiotherapy; Chemo, chemotherapy. ∗ This patient was diagnosed with Li-Fraumeni syndrome.

Radiation-induced DNA damage and genomic instability constitute the core pathogenesis of RIS. Ionizing irradiation initiates DNA double-strand cleavage and reactive oxygen species accumulation, which in turn provoke extensive genetic alterations. Meanwhile, the radiation-induced bystander effect increases the mutational vulnerability of non-irradiated neighboring cells, providing a plausible biological explanation for the protracted latent period of RIS development (8, 18, 19). Furthermore, RIS is also closely related to multiple factors, such as chronic lymphedema, young age at radiotherapy, radiation dosage and delivery technique, concurrent administration of alkylating or anthracycline chemotherapy, and hereditary tumor predisposition syndromes (4, 8, 20, 21).

TP53 is the most commonly mutated gene in RIS, with a mutation rate of 58% (22). It has been reported that TP53 gene abnormalities are correlated with the advanced clinicopathological stage and poor prognosis of leiomyosarcoma (23). Leiomyosarcoma is inherently insensitive to conventional chemotherapy, and concurrent TP53 mutation further compromises responsiveness to doxorubicin- and ifosfamide-based regimens, readily leading to chemoresistance and treatment failure. In addition to genetic defects, PD-L1 expression status is a critical determinant of immunotherapy efficacy in soft tissue sarcomas. Leiomyosarcoma exhibits intrinsically low PD-L1 expression and represents the histological subtype with the poorest response to PD-1/PD-L1 blockade, with an objective response rate as low as 6.9% (24). Low intratumoral T-cell infiltration and an immunosuppressive microenvironment further weaken the antitumor immune response of leiomyosarcoma, limiting the clinical benefits of immune checkpoint inhibitors. In addition, this tumor has a high degree of malignancy and strong proliferation and invasion abilities. The tumor blood supply is complex with multiple collateral circulations, making it difficult to completely block the tumor blood supply through simple transarterial chemoembolization. Collectively, the coexistence of high-frequency TP53 mutation and low PD-L1 expression characterizes a therapy-resistant molecular subtype of radiation-induced pulmonary leiomyosarcoma, accounting for its refractory clinical course and dismal prognosis.

Apart from TP53, alterations in other key genes also exert potential roles in RIS pathogenesis. MYC amplification is nearly universal in radiotherapy-associated angiosarcoma but rarely detected in other RAS histological subtypes, and its overexpression alone cannot confirm a radiation-related origin (25). Existing genomic studies have suggested a possible link between BRCA mutations and RIS susceptibility (8), though relevant conclusions remain controversial (26).

Clinically, leiomyosarcoma may present as nonspecific soft-tissue thickening, a painless mass, or painful changes in a previously irradiated area. Given these non-specific clinical presentations, radiological imaging plays a key role in the early detection, differential diagnosis and longitudinal surveillance of RIS. Typical imaging manifestations include soft tissue mass formation, adjacent bone destruction, tumor necrosis and heterogeneous enhancement, which lack absolute specificity but possess important warning value (8, 27). If the above radiological features emerge within the radiation field, RIS should be highly suspected. Unlike conventional primary lung cancer, RIS rarely manifests with spiculation, lobulation or pleural traction. In contrast to infectious lesions, it shows no signs of dynamic inflammatory resolution or cavity formation. For radiologists, regular long-term thoracic CT follow-up is essential for breast cancer patients post-radiotherapy. Special attention should be paid to newly developed soft-tissue masses with rapid growth, even more than 20 years after irradiation. Therefore, regular follow-up after radiotherapy is indispensable. Zhang et al. recommended breast X-ray and non-contrast computed tomography of the chest every 6 months during years 1–7 after breast cancer radiotherapy, then annually (10). However, in patients with TP53 mutation and breast cancer, non-contrast computed tomography of the chest follow-up should be considered to reduce the risk of RIS. Several guidelines recommend annual whole-body diffusion-weighted magnetic resonance imaging after radiotherapy to monitor for RIS (28).

Until now, no standardized therapeutic regimen has been established for RIS due to limited clinical data. Surgical resection with 2–3 cm wide negative margins remains the first-line curative option, and R0 radical resection confers significantly better overall survival than conservative management (2, 8, 25, 29). Anthracycline-based adjuvant chemotherapy yields certain clinical benefits but remains controversial owing to limited evidence (4). Re-irradiation is restricted to selected cases due to severe toxicities, with unproven prognostic superiority (10). Neoadjuvant chemotherapy can downstage tumors and facilitate R0 resection, while PD-1/PD-L1 immunotherapy has shown promising efficacy in selected RIS subgroups (22). In the future, further investigations into the molecular and genomic features of RIS are warranted to identify reliable prognostic biomarkers and actionable therapeutic targets.

## 4 Conclusion

RIS following breast cancer surgery is a rare late radiotherapy complication with an ultra-long latency and variable individual prognosis. For such high-grade RIS subtype harboring TP53 mutation and low PD-L1 expression, intrinsic resistance to chemotherapy, PD-1 immunotherapy and anti-angiogenic therapy commonly results in multi-modal treatment failure. Radical surgery remains the only curative option, while empirical multi-line therapy provides limited benefit for unresectable elderly cases, for which individualized palliative care is preferable to maintain quality of life. Radiologists should be alert to rapidly enlarging pulmonary masses arising within the radiation field among long-term breast cancer survivors, facilitating early identification and avoiding misdiagnosis. More case reports and multi-center studies are needed to accumulate clinical data and formulate standardized diagnosis and treatment protocols for this rare tumor.

## Data Availability

The original contributions presented in the study are included in the article/supplementary material. Further inquiries can be directed to the corresponding authors.
